# **BAROS METHOD CRITICAL ANALYSIS** (BARIATRIC ANALYSIS AND REPORTING SYSTEM)

**DOI:** 10.1590/S0102-6720201500S100020

**Published:** 2015-12

**Authors:** Jean Ricardo NICARETA, Alexandre Coutinho Teixeira de FREITAS, Sheyla Maris NICARETA, Cleiton NICARETA, Antonio Carlos Ligocki CAMPOS, Paulo Afonso Nunes NASSIF, João Batista MARCHESINI

**Affiliations:** Surgery Department, Federal University of Paraná, Curitiba, PR, Brazil

**Keywords:** Obesity surgery assessment, Co-morbidities, Life quality, Complications

## Abstract

**Introduction:**

: Although it has received several criticisms, which is considered to be the most effective method used for global assessment of morbid obesity surgical treatment, still needs to be updated.

**Objective:**

: Critical analysis of BAROS constitution and method.

**Method:**

*:* BAROS as headings was searched in literature review using data from the main bariatric surgery journals until 2009.

**Results:**

: Where found and assessed 121 papers containing criticisms on BAROS constitution and methodology. It has some failures and few researches show results on the use of this instrument, although it is still considered a standard method. Several authors that used it found imperfections in its methodology and suggested some changes addressed to improving its acceptance, showing the need of developing new methods to qualify the bariatric surgery results.

***Conclusion*:**

BAROS constitution has failures and its methodology needs to be updated.

## Abstract (pt)

**Introdução:**

: O BAROS é considerado o método mais eficaz e utilizado para a avaliação global do tratamento operatório da obesidade mórbida; porém, possui inúmeras críticas e precisa ser atualizado.

**Objetivo:**

: Analisar criticamente a constituição e o método do BAROS.

**Método:**

: Revisão da literatura utilizando o descritor BAROS, pesquisado nas revistas de cirurgia bariátrica até abril de 2009.

**Resultados:**

: Foram encontrados e avaliados 121 trabalhos. O BAROS possui falhas em sua constituição e metodologia. Embora ainda seja considerado como método-padrão, poucas pesquisas relatam resultados utilizando esse instrumento. Inúmeros pesquisadores que utilizaram este protocolo encontraram imperfeições em seu método e sugeriram modificações para que fosse amplamente aceito, tornando-se evidente a necessidade de um novo método para qualificação dos resultados das operações bariátricas.

**Conclusão:**

: O BAROS possui falhas em sua constituição e necessita de atualização em seus meios metodológicos.

## INTRODUCTION

BAROS (Bariatric Analysis and Reporting Outcome System) was developed with the aim of globally assessing and trying to make the reports on the bariatric surgeries be uniform, and nowadays it is the principal instrument used by the medical societies in order to report the results of these surgeries^16^.

Although its use is widely encouraged, few researches report results from the use of this instrument^1 ^and countless researchers report flaws in its composition and find difficulties in its application, and they criticize the methodology. Because of this, it is believed that some of its criteria must be reassessed and updated, bringing about the evidence that there is the need for the creation of a new method to qualify the bariatric surgery results. 

Thus, this article aims to perform the critical analysis of the constitution and BAROS method.

## METHOD

Literature review using the headings "BAROS, avaliação da cirurgia de obesidade. Comorbidades, qualidade de vida, complicações", by searching all in the database online of the principal journals on bariatric surgery up to April 2009^14^.

## RESULTS

One hundred and twenty-one papers were found and assessed, and all the reviews on the use of this method were analysed and reported.

Facing the difficulty to measure the result of the bariatric surgeries in a simple and effective way, Oria e Moorehead introduced BAROS. It had the aim to globally assess and try to standardise the report on the bariatric surgery results. Five principal aspects were pointed out: weight, comorbidity, life quality, complications and new surgeries. The final score classifies the result into five groups: excellent, quite good, good, acceptable and insufficient^16^^,^ 17.

BAROS is the principal method to globally assess the results of the bariatric surgeries; however, in its clinical application, countless failures in conception and constitution were observed, and they made the analysis, comparision and statistic interpreation of data difficult^5^. Responding to countless criticisms from researchers, BAROS was reformulated, principally in relation to life quality, what brought about the development of Moorehead-Ardelt II Questionnaire. It adds a sixth question about the patients' eating behavior and the analysis method was restructured as well. In spite of the changes, the questionnaire did not correct all the imperfections, and it keeps on not being applicable to all persons^11^.

Searching for BAROS descriptor on the internet, on the principal journals that publish articles on this theme, only 121 papers made use of it, showing that, although it is recognized as an assessement tool for the bariatric procedures, it is scarcely used, what indicates that its methodology needs to be reviewed^5^^,^ 14.

### General aspects of the composition and adequate method for the assessement to bariatric surgeries

For the consensus of the USA National Institute of Health of 1991^13^ as well as for Oria, Mason and Deitel^15^, there are failures in the assessement of the bariatric surgeries, once the analysis of the results needs to observe all the spectrum involved in the treatment of morbid obesity. It is important that long term effects from the diseases and the surgical treatments are determined, contributing in this way, to the perfection of the art.

BAROS was studied, and in practice, it does not allow analysing properly the pre and postoperative of the several interventions - clinical, surgical or endoscopy related - performed for the obesity treatment. It is not able to collect and assess all the changes that occur with the patients who undergo surgery, since the pre to the remote postoperative and, besides, it does not standardize the prospection and analysis of results, preventing in this way its use to compare the multiple aspects involved.

The analysis of the bariatric surgeries results, as well as similarly, the concept of health, involve multiple variables and can not be defined through mere scoring of complications and weight reduction. Thus, in order to perform the analysis it is necessary to have a multi-dimensional constructo, assessing all the aspects involved in the health concept.

In the analysis of the bariatric surgeries, the health condition must be collected and measured in an objective scale; in other words, the better it is, the greater the final result will be; the worse it is, the lesser it will be.

BAROS does not allow the comparison of the health condition of the pre and postoperative, and the patient is not at his own control. It places different patients in equal groups, limiting in this way, the capacity for data prospection. It uses qualitative data in each domain and the final result is divided into five subgroups. The values are expressed in attributes (for example: improved or worsened comorbidity, or good, quite good or excellent). It sets patients having very different characteristics and results into the same group, making them equal and grading them in the same way, limiting thus, the capacity for differentiation. This grading way, besides being very confusing, makes it difficult to establish the statistic analysis, and consequently reduces the accuracy of the method. 

A method which has a high capacity to differentiate the results from different persons must be used in the analyses of bariatric surgeries results, preventing in this way, the grouping of different patients in the same category. It must provide unique and individual grading, in consonance with the current concept of health, allowing the pre and postoperative prospection and comparison in any kind of intervention.

### Weight assessment through BAROS

The assessment of weight loss through BAROS uses the weight excess reduction percentage (%EWL), index derived from the ideal weight, which bases itself on the population study of the Metropolitan Life Insurance Company^10^. There are several statements to this table and several irregularities occurred in the collection of its database: they were collected in 1979, and now the people live more and the ideal weight associated to the the highest longevity has increased; the researched population was disproportionaly higher in white people; the weights were self informed in 10% of the clients; the insured population corresponded to a very high socioeconomical group; the weights were taken into consideration when people were wearing light clothes and shoes; persons who had a stroke, cancer, diabetes and hypertension were excluded; the average weight considered was from persons who were 25-59 years old. Besides, the ideal weight may not be applicable to all the population, principally in the XXI Century, running the risk of being inappropriate for the assessment of morbid obese person, when losing weight. So, in spite of the fact that %EWL had been widely used, nowadays it is considered as having lost its accuracy^3^^,^ 4 ^,^ 6 ^,^ 19.

The OMS uses the body mass index (BMI) as pattern to describe, classify and compare obesity, because it has good accuracy, is largely used and calculated. The result of the BMI is applicable to all the persons and populations, has direct relation with the mortality and comorbidity rates, high correlation with the body density and skin folds, being a good sign of adiposity. It is of easy interpretation in pre and postoperative situation and it gives the real dimension of the weight reduction after the treatment. It is not subject to the possible errors related to the Metropolitan table of ideal weight^4^.

For Sánchez-Cabezudo e Larrad^20^ e Deitel e Greenstein^4^, the changes of weight excess and of the BMI are better ways to assess the evolution of the weight, and they recommend the use of the %EWL, IMC e %EBL for the postoperative follow-up. However, for Dixon, Mcphail e O'Brien^6^, the %EBL should be adopted as standard, what suggests that poor definitions, such as ideal weight and its derivations, were abandoned. It was for these reasons that, according to Deitel e Greenstein^4^ e Deitel, Gawdat e Melissas^3^, the journal Obesity Surgery adopted the Percentage of Excess of Loss (EBMIL or %EBL) as standard to report on the variation of weight in the postoperative concerning the bariatric surgeries.

The stratification of the result of the weight loss in five groups is another criticism to BAROS. When grouping the result into breaks, it changes itself from a continuous variable to a qualitative one, making the statistics analysis difficult. The grading of this item is based on the categorization of groups with breaks of 25%. In this way, it classifies the person who lost 25% into the same group as the one who lost 49% of the weight excess, being both graded as "more 1 (+1)". This difference is obviously significant among the patients, and probably the result of the analysis of the other factors will show it. Going deeper into the analysis, it is clearly observed that these patients are different and can not have the same kind of grading.

### Assessment of the comorbidities through BAROS

The improvement of the comorbidity is the principal result of the bariatric surgeries. For this reason, the evolution in the postoperative is an important factor for the assessment of the success of the procedures and such assessment must be done in the most effective way possible^12^.

On BAROS the assessment of the comorbidities has many imperfections and no article searched supplies detailed data on this important aspect. It is done in an isolated and limited way, studying the effect of the surgery on one or two diseases, and not analysing the alterations of multiple diseases in a global way. It groups different diseases and evolutions into the same category. Its grading is based on inaccurate qualitative data, being then confusing and superficial (a solved comorbidity=+ 2 points or all the solved comorbidities =+ 3 points). This datum brings little information about the real effect of the procedures and it does not translate its importance to the improvement of the postoperative morbidity. 

On BAROS, the assessment of the alterations of the comorbidities in the postoperative stage does not differentiate the patients having or not having diseases before going through the surgery. The ones classified as not having any alteration in the comorbidities may be patients who did not have diseases in the preoperative stage or who had them at this stage, but did not present any improvement after the surgery. These two groups of patients are different and can not be assessed together.

On the present method, patients who did not have comorbidities before going through the surgery will score lower (0 point) than those who had the comorbidities before the surgery. So, the group who has the highest number of patients without diseases in the preoperative stage, will have the lower average grading (score) at this stage. On the other hand, the group who has the highest number of comorbidities per patient in the preoperative stage will have the highest grading (score) concerning this matter. The lowest grading (score) in the alterations of the clinical conditions results in worse results in the final assessment of BAROS. It prevents the isolated use of the alterations of the comorbidities to compare different groups of patients and surgery techniques. When getting the final result on BAROS, it is tried to partially correct the distortions in the assessment of the comorbidities whenever they classify the patients as "having" or "not having" comorbidities using scales of different points. It mixes the methodology up even more and does not solve such inaccuracy.

Another criticism to BAROS happens due to the fact that many diseases existing in the patients (minor comorbidities) are not assessed by Oria e Moorehead, such diseases may affect their lives in a varied way. The consequence is that many comorbidities are not used for obtaining the score at the domain of the clinical conditions, what makes this method little accurate and unable to analyse all the spectrum of the diseases. It is important to point out that patients having the diseases, such as acid reflux, may have worse life quality than the ones having heart disease or hypertension.

In the patients having diseases not assessed by Oria e Moorehead, the postoperative weight loss may cause considerable improvement to these comorbidities. This fact, besides influencing the score of this stage, may interfere in other BAROS's domains, such as life quality.

On BAROS there is no consensus on the resolution, improvement or worsening of the diseases, and not even on each way to show these variations. The criteria used by Oria e Moorehead^16^ are different from those described by Melissas *et al.* 9 They show the difficulty to standardize the reports on the alterations of the comorbidities at the postoperative stage of the bariatric surgeries.

Victorzon e Tolonen^22^ criticize two aspects of the assessment of the clinical conditions on BAROS. They state that there are different diagnostic criteria for each disease and that the definition of the improvement or resolution of each disease associated directly or indirectly to obesity is not clear. They conclude that this aspect must be better studied and, if possible, aggregated to BAROS for it to be more widely accepted, since it does not allow to compare the alterations of the comorbidities in different techniques and scientific studies properly.

In relation to the comorbidities, BAROS's categories are confusing and the pieces of information transmitted by them are not clear and are not subject to deeper analyses. Providing this classification, it is difficult to estimate what really happens to the patient, once several possible therapeutic answers are grouped together (resolution, improvement, worsening, so forth) of different diseases (HAS, dyslipidemia, among others) at the same group, as for example, that one characterized as "one of the biggest co-diseases solved and others improved". The information extracted from this classification does not allow making the therapeutic answer be individual for each disease facing the treatment of the morbid obesity through surgery.

A big number of co-diseases - as for example the acid reflux - is not considered, not allowing in this way, the individual analysis of all the diseases due to its grouped results. Thus, studies that are based on this method can not analyse the evolution of the comorbidities deeply; so, it is necessary to find an adequate way to assess the real changes in the comorbidities after the bariatric surgeries, because it is not worth reporting the weight loss properly without informing the improvement of the comorbidities in details.

BAROS should include all the possible diseases that the patients may develop, being them more serious or minor, related with obesity or not, and not only the diseases listed by Oria e Moorehead. All patients should be assessed in an equal way, preventing the differentiation that BAROS makes between persons having and not having comorbidities, independently from the clinical conditions of the pre and postoperative stages. 

In order to improve the analysis of the diseases it is important to standardize the comparison of the alterations of the comorbidities after the bariatric surgeries. It is necessary to give details and specify whether there was resolution, improvement, worsening or no alteration of all the diseases that the patient had before undergoing the surgery.

It is necessary to define more objectively how to classify the condition of each disease and its evolution since the preoperative up to remote postoperative, including the individual analysis of the condition of each disease, avoiding the classifications used on BAROS, such as "no alteration", "improved", "worsened".

### Assessement of the morbimortability through BAROS

No method, including BAROS, analyses the relationship between morbimortability risk, adiposity, obesity, and not even its evolution in the postoperative stage of bariatric surgeries. 

The morbid obesity may be considered as a mixed risk factor (acquired, environmental and genetic), being substrate for the development of countless other comorbidities. The association of multiple diseases and risk factors is the rule and not the exception, increasing considerably the rates of morbimortability in relation to the population that is not obese.

A relevant aspect for the morbid obese people is the fact that many of them are considered healthy for not presenting any associated disease. In these cases, the simple assessment of the effect of the operation in the comorbidities can not be applied. These patients, however, in the evolution of their diseases, may present countless other comorbidities; therefore, it is important to evaluate which is the effect of the operation and how it may intervene in the health-disease process.

The quantification of the risk and the setting of the proper prognostic or of risk bands for a specific patient or doctor's action are, maybe, some of the most difficult tasks in the medical practice.

The risk of morbimortability, according to authors, should be studied and, if possible, aggregated to BAROS, being this one, one of the principal criticisms to such method.

Victorzon e Tolonen^22^ called the attention to the reduction of the risk of development of diseases in obese patients who underwent surgery. Deitel, Gawdat e Melissas^3^ e Baltasar, Deitel e Greenstein^1^ stated that the assessment of the adiposity is essential for the analysis of the surgical results and they raised the hypothesis of including the assessment of morbimortability risk for the morbid obese people.

There are few papers that study, in details, the effect of the bariatric surgery on risk factors and their relationship with the health-disease process. Not long time ago, for example, it was thought that the improvement of the diabetes was due only to the losing of weight; however, new studies showed that the surgery induces changes in the metabolism of the glucose (rubin effect, incretin effect), such changes are the principal ones responsible for the improvement^7^^,^ 8 ^,^ 12 ^,^ 18. Thus, the assessment of the health-disease process is of essential importance through the analysis of the comorbidities and risk factors of morbimortability on the new BAROS.

The bariatric surgeries act in multiple aspects, resulting in marked alteration or elimination of several factors of morbimortability risk, changing, in this way, the natural history of several diseases, not only for preventing them from developing (primary prevention), but also, for preventing or reducing the risk of adverse events (secondary and terciary preventions). The inclusion of risk rates on BAROS would measure how much the surgery can change (eliminate or reduce) the probability of some persons coming to develop diseases or die from them, inaugurating in this way, new study border on the treatment of this disease.

The analysis of the morbimortability on BAROS must base itself on the rates and indicators accepted worldwide, and which are able to identify and stratify the risk, allowing, in this way, to implement strategies for decreasing the morbidity and death in morbid obese people. It should include a measure that was missing for the assessment of the surgical results and it would inaugurate a new paradigm in the analysis of procedures. 

There are few papers that show the reduction of the girth of obese people who underwent surgery. Carvalho *et al*.^2^demonstrated that there is significative reduction of the girth after the bariatric surgery; however, it still remains above normal. They concluded that more studies are necessary concerning the assessment of the impact of these procedures in the anthropometric measurements in order to establish new cutting points for this population.

To end up the discussion on this theme, the anthropometry, in special, the girth, must be used to estimate the risk the morbid obese person runs to develop diseases before and after the surgery. As shown by the literature, when the patient loses weight after the bariatric surgeries, there is improvement of these rates and, as a consequence, there is reduction of the morbimortability, principally the one related to the diseases concerning the cardiovascular system and metabolic syndrome. The adiposity has straight correlation with the morbimortability, however, BAROS does not assess this parameter. For many authors, it would be interesting to report the adiposity in the analysis of the results. Among the objectives of this operation are the improvement, cure and prevention of the several diseases that attack the morbid obese people, reducing thus, the risk of morbidity and mortability, and for this reason, such datum should be better studied and incorporated to the analysis of the results^1^^,^ 16.

The data of the literature support, nevertheless, the incorporation of the risk assessment of the morbimortability in the bariatric surgeries for bettter understanding.

### Assessment of the life quality through BAROS

Life quality is the principal question to determine the final result of BAROS and one of the principal criticisms to Moorehead-Ardelt questionnaire. In specific situations, it may not be applied to all the patients, as for example, the sexual activity in celibate and elderly people, and physical activity and capacity to work with patients who are disabled or elderly. In these persons, the grading of life quality and as a consequence, of BAROS will be smaller.

The question about life quality, especially the one about self-esteem, is the item which influences the final result of BAROS the most, being dependent on the psycho-social condition of the patient, since depressed persons have lower scores when compared to the ones having normal psychological condition. It suggests then, that the new studies should be done for improving this item of the method.

The morbid obese people are holders of multiple co-diseases that affect them in a varied way. These diseases may be partially or totally controled with bariatric surgery; however, it does not necessarily results in having better life quality.

When it is intended to determine the impact of the interventions on the most affected people, it is necessary to assess their experience through the subjective assessment of life quality, aspect which is intimately related to one of the basic human being's wishes: living life and feeling well. Before this new paradigm, the assessment of life is the measurement that was missing in the health area^ 24^.

The definition proposed by the Life Quality Group from the sector of Mental Health of OMS is the one that best translates the reach of the life quality construct, being understood as: "the person's perception of one's position in life, the context of one's culture and in the system of values where one lives in relation to one's expectations in life, life standards and worries ". It considers life quality in a very wide sense, which incorporates, in a complex way, one's physical and psychological health, level of dependence, social relationships, personal beliefs and the relationship with significant aspects of the environment^24^.

Several methods may be used in order to measure the perception/sensation of the human being's feelings. Many of them are applied to the life quality of bariatric patients,; however, these questionnaires, at least great part of them, are long, very sophisticated and are not designed especifically to morbid obese people undergoing surgeries for weight reduction. Besides, they are scarcely answered in a proper way many years after the surgery took place^15^^,^ 16 ^,^ 17 ^,^ 24.

As the morbid obese people present several comorbidities and multiple interrelationships among them and the life quality, it seems to be clear that a generic instrument must be employed, one which may collect all the spectrum of diseases and their influence on the person's health condition. In this way, very specific questionnaires may get all the possible variations in the life quality of this population. Therefore, the most recommended is probably the use of a multidimensional object, which is wide and adapted to the nuances of the bariatric surgery.

Moorehead-Ardelt questionnaire of BAROS is the most used for the assessment of the bariatric surgeries; however, it is criticized by many authors, and several studies show that it is not able to assess morbid obese people's life quality properly. It is not able to assess the perception of life quality in each patient in a global and individualized way. Besides, the eating or digestive habits have fundamental importance in the composition of morbid obese people's life quality, and this fact is neglected by the questionnaire.

Many patients report significant improvement in their humor after the weight reduction, and some euphoria after the surgery. The improvement of the psycho-social aspects may overestimate life quality's improvement and influence the result of BAROS. The overestimated value of life quality increases the score of BAROS, being likely to impair the efficiency of the method. On the other hand, the depressed people report the worst levels of life quality. It results in different perceptions on health improvement, and for this reason, psycho-social aspects may overestimate or understate life quality improvement and influence BAROS results^13^^,^ 21 ^,^ 23.

Moorehead *et al.* 11 replying to countless researchers that used BAROS, tried to polish the initial questionnaire on life quality and developed the Moorehead-Ardelt II questionnaire. Despite the changes adopted, they did not correct all the imperfections of the method, once this instrument isn't applicable to all the patients yet, it does not allow the individual building-up of the life quality construct, and it continues being highly influenciable by the psycho-social state (condition).

Moorehead-Ardelt Questionnaires I and II, hence, do not analyse all the aspects involved in the treatment of obesity and do not assist the life quality concept adopted by OMS. For these reasons, they seem not to be the most adequate to assess the wide spectrum of changes that occur with the morbid obese patients after they undergo surgery.

Among the peculiarities of morbid obese people it is the eating habit, once, many times, such patients "live to eat". The way one eats is the principal change that occurs, and everybody knows that many patients do not adapt to the new reality and start having eating disorders and difficulties, such as dysphagia, vomiting, dumping, among other symptoms. For this reason, such aspect needs to be included in the assessment of patients' life quality, mainly for the persons who underwent restrictive surgeries.

Disabsortive techniques may cause important changes in intestinal habit. Many patients may present uneasy symptoms, such as profuse diarrhea and flatulence with strong smell, what may also mean worse life quality after undergoing the surgery.

As the intestinal and eating habits may interfere in the surgical result and, consequently, in the patients' life quality, such peculiar aspects of morbid obese people support changes in BAROS, including mattters referring to the digestive system.

### Complications and reoperations

BAROS classifies the persons independently from the number of complications and new surgeries they had undergone. In the bariatric surgeries, the bigger the number of complications and new surgeries, the greater the surgical risk is, what justifies the reformulation of this domain.

In any treatment it is of essential importance to assess all the possible complications related to it. Efficient therapy is not enough, but it must also have low rates of complications that may alter its benefits.

In the analysis of the bariatric surgery, the complicatons and new surgeries may interfere with the weight loss, changes of the comorbidities, life quality and postoperative therapeutic success, acting directly on the risk of morbimortability. The higher the number of complications and new surgeries, the higher the surgical risk of therapeutic failure, and the more elevated the mortality rate of the surgical treatment of obesity will be. It is no use the surgery to have excellent results concerning the weight loss and the improvement of the diseases if the surgery morbimortality is high. 

On BAROS the complications may be classified into surgical and clinical, major or minor, early or late, including almost all the diseases related to the procedures; however, many surgeries that take place as a consequence, such as functional cholecystectomy and dermolipectomy, may have great impact on the global surgical risk.

BAROS' scoring is defined by the type of complication that occurred, and three types of different scores are possible: without complications (0 point), minor complication (-0,2 points), major complication (-1 point), independently from the number of injuries that occurred, receiving the highest scoring of -1,2 points. The new surgeries, on the other hand, may receive two possible classifications: with new surgery (-1 point) and without (0 point). The summing up of the complications and reoperations may vary from 0 to -2,2 points.

According to the present methodology, it does not matter whether the patient had one or more major or minor complications and, even, if he underwent a new surgery once or more times; the score will always be the same. Besides, in case a complication results in reoperations, the scoring of the first is not considered in the analysis of this domain. It distorts the assessment of this important surgical aspect, because there is no direct correlation between the number of complications and the new surgeries that took place and, not being possible to identify, in a proper way, which technique presents minor surgical risk.

Patients that had one or more complications, or those who needed one, two or three new surgeries, can not score equally. The higher the number of complications and new surgeries, the higher must the score of this domain be, which will act negatively in the final result of the analysis.

From the clinical point of view, knowing the number and how serious the complication is as well as the new surgeries is fundamental for the results. Having more capacity of prospection and analysis of this important aspect justifies the need for the reformulation of BAROS.

It is necessary to assess all the complications and reoperations that took place, generating unique score, which will be higher according to the number of diseases observed; in other words, the higher the number of complications and new surgeries, the higher the surgical procedure risk is, therefore, the score of this item must be higher.

### Final result of BAROS

The observation of variable must generate one, an only one result. Whenever a feature can be properly measured under the quantitative way (numerically), there is great gain in terms of techniques of exploratory analyses of data, and preference must be given to this kind of mensuration.

Countless methodological distortions take place in BAROS, since, as it has already been described, it bases itself on innaccurate qualitative data and has limited capacity to assess the results of the bariatric surgeries, making the correct comparison between the different methods and patients impossible, and running the risk of making the analysis of the final result through this instrument be completely invalid.

BAROS stratifies the domains and the final result into five groups, classifying the persons with different results into the same category, and it does not allow the comparison of the data of the preoperative with the postoperative, what makes the statistics analysis of the results difficult. There is great distortion of the final result, since patients with or without comorbidities are scored differently, and the final scores of these two groups are not comparable. The persons with diseases in the preoperative may get to the highest score of nine points, and those without comorbidities may get up to six points. Then, the persons with comorbidities in the preoperative will always have higher score than those without them. Not even the mathematical solution applied to this method, which is to deduct three points from the persons who have comorbidities, is able to correct this distortion; instead it mixes up the analysis of the final score even more.

### Final considerations

In this paper countless distortions of BAROS were found out and it was proved that it is unable to collect all the aspects involved in the treatment of morbid obesity, what limits the analysis of the surgeries, and what justifies the methodological review of the instrument.

It is necessary to correct BAROS, by improving the method of prospection and analysis of data in all domains assessed, and studying, in details, the multiple aspects related to morbid obesity and its treatment.

Its reformulation, or better saying, the new BAROS, must allow the assessment of the bariatric surgeries in a more clear, objective, broad and deep way, and be able to be applied and reproduced in all studies, in a transcultural way, collecting the nuances of this complex disease, and standardizing the data report, allowing all the surgeons to be able to speak the same language with the final aim of perfecting and improving the results of these surgeries.

## CONCLUSION

By analysing BAROS in a critical way it was possible to clearly notice its failure in the collection of all the aspects involved in the treatment of the morbid obesity, since failures are pointed out in its constitution, what limits the analysis of the bariatric surgeries through this method. It justifies, in this way, the reassessment and reformulation of the methodology of this instrument, aiming to perfet and improve it and make it more efficient and able to be broadly used in the assessment of the result of the bariatric procedures.
